# Mixed cryoglobulinaemia vasculitis secondary to marginal zone lymphoma in a patient with end-stage renal failure on haemodialysis

**DOI:** 10.1007/s13730-023-00823-5

**Published:** 2023-10-06

**Authors:** Craig Peter Coorey, Amirhossein Aarabi, Karthik Kumar

**Affiliations:** 1 Gosford Hospital, Gosford, New South Wales Australia; 2 School of Medicine, Western Sydney University, New South Wales, Australia

**Keywords:** Lymphoproliferative disease, Vasculitis, Cryoglobulinaemia, Haemodialysis, Chronic kidney disease

## Abstract

Cryoglobulinaemia vasculitis can present with a variety of symptoms and there is limited data on the incidence and presentation of cryoglobulinaemia vasculitis in haemodialysis patients. We report a case of a 63-year-old male who had a series of presentations with rash, visual changes, abdominal pain, weight loss, fevers and digital ischaemia. This is on a background of a congenital single kidney with end-stage renal failure secondary to diabetes and hypertension, receiving haemodialysis for nearly 5 years. He initially experienced a leukocytoclastic vasculitis rash confirmed on skin biopsy, followed by multiple hospital presentations for undifferentiated abdominal pain and fever of unknown source. Jejunal biopsy revealed intestinal vasculitis. His peripheral blood flow cytometry and bone marrow biopsy were consistent with marginal zone lymphoma (indolent subtype, IgM kappa clone). Further testing revealed a type II cryoglobulinaemia consisting of an IgM kappa monoclonal band with polyclonal IgG (cryocrit 5%). A diagnosis of cryoglobulinaemia vasculitis was established and he was treated with pulsed methylprednisolone and rituximab therapy. However, after receiving three doses of rituximab the patient developed a presumed vasculitis-associated pulmonary haemorrhage for which he received treatment with five sessions of plasma exchange. His symptoms resolved and cryocrit reduced to < 1% after his final dose of rituximab. The clinical features of cryoglobulinaemia may be difficult to detect in chronic haemodialysis patients and vigilance is required.

## Introduction

Cryoglobulinaemia vasculitis is a small-vessel vasculitis defined by the presence of cryoglobulins that precipitate at temperatures cooler than core body temperature. Cryoglobulinaemia is typically classified as either type I, type II or type III depending on its immunoglobulin (Ig) constituents [1]. Type I cryoglobulinaemia comprises monoclonal Ig (either IgM, IgG, or IgA) and is usually associated with B-cell lymphoproliferative disorders such as lymphomas and multiple myeloma. In contrast, the cryoglobulins in both type II and type III cryoglobulinaemias are mixed, comprising more than one type of Ig. Type II cryoglobulinaemia is typically characterised by polyclonal IgG and monoclonal IgM, whilst in type III cryoglobulinaemia, there are immune complexes formed by polyclonal IgM and polyclonal IgG. Type II and type III cryoglobulinaemias are commonly associated with infection (particularly Hepatitis C), B-cell malignancies and autoimmune conditions such as primary Sjogren syndrome and systemic lupus erythematous. Patients with a mixed cryoglobulinaemia who do not have a clear underlying cause are classified as having an essential mixed cryoglobulinaemia.

Cryoglobulinaemia vasculitis can have a varied presentation involving multiple body systems [2]. It commonly involves the skin (usually with a palpable purpura) but can involve the nervous system, kidneys, joints, gastrointestinal tract, heart and lungs. The disease severity also can range from mild symptoms to life-threatening disease. Non-specific symptoms such as fatigue are often also present.

## Case report

We present a 63-year-old male with a background of congenital single kidney and end-stage renal failure on haemodialysis presumed secondary to hypertension and type 2 diabetes mellitus. He commenced haemodialysis 5 years ago. His background history included ischaemic heart disease with multiple coronary artery stents (most recent stents inserted 3 years ago), dyslipidaemia, right parietal stroke (at age 56 years old), gout, obstructive sleep apnoea and previous tuberculin test positive that was treated with standard three-drug treatment for 3 months.

A summary of his presentations is presented in Table 1.

**Table 1:** Summary of key events and results. MRI: magnetic resonance imaging

Timepoint	Event
October 2019	Leukocytoclastic vasculitis rash on legs. Treated with corticosteroids
March 2021	Clinical temporal arteritis with negative biopsy. Treated with corticosteroids MRI brain scan showed cerebral vascular calibre irregularities
June to July 2022	Multiple presentations with abdominal pain. Treated as acute cholecystitis and small bowel enteritis based on imaging findings Further episode of visual loss. Treated with corticosteroids C4 level 0.09 g/L (ref 0.15 to 0.53)
August 2022	Readmitted with abdominal pain, weight loss and fevers. Raised inflammatory markers: C-reactive protein > 200 mg/mL (ref < 5) and procalcitonin 2.75 ug/L (ref < 0.05) Imaging demonstrates new hepatosplenomegaly Diagnosed with indolent marginal zone lymphoma
September 2022	Leukocytoclastic vasculitis rash on legs Digital ischaemia Jejunal biopsy demonstrating intestinal vasculitis Serum cryoglobulins positive (cryocrit 5%) and C4 level 0.03 g/L (ref 0.15 to 0.53) Diagnosed with type II cryoglobulinaemia vasculitis, likely secondary to marginal zone lymphoma. Commenced corticosteroids and rituximab. Cryocrit reduces to 2% after treatment commencement
October 2022	Vasculitis-associated pulmonary haemorrhage. Cryocrit rises to 3%. Treated with plasma exchange After final dose of rituximab, cryocrit reduced to < 1% and C4 level increased to 0.75 g/L (ref 0.15 to 0.53)

Two years following the commencement of his haemodialysis, he developed a non-blanching purpuric rash on his lower limbs with a skin punch biopsy demonstrating a small-vessel leukocytoclastic vasculitis that was successfully treated with a short course of oral prednisolone. Two years later, he subsequently experienced right-sided headaches, blurred vision and jaw claudication that was investigated with a right temporal artery biopsy revealing normal histopathology. However, his magnetic resonance imaging (MRI) brain scan (without contrast) showed cerebral vascular calibre irregularities, suggestive of cerebral vasculitis or intracranial atherosclerotic disease. He was treated as presumed giant cell arteritis with oral prednisolone and had resolution of his symptoms.

One year later, he had multiple presentations to hospital with generalised abdominal pain, fever and rigors without vomiting or diarrhoea. On the first presentation, his computed tomography (CT) abdomen scan showed soft-tissue stranding around the gallbladder and within the gallbladder fossa consistent with acute cholecystitis. He was medically managed with intravenous antibiotics. He also had an episode of headaches, conjunctival injection and blurred vision for which he received a single dose of intravenous methylprednisolone and symptoms promptly resolved. On his next presentation with abdominal pain, his CT abdomen scan showed mural thickening of the small bowel loops (in particular jejunum), with adjacent inflammatory stranding and mesenteric free fluid, suggestive of small bowel enteritis. He was treated with intravenous antibiotics with partial resolution of his symptoms. No pathogenic organisms grew on faeces culture. His blood test results at the time are summarised in Table 2.

**Table 2:** Summary of blood test results during the patient’s presentations with abdominal pain. RNP: ribonucleoprotein, PCNA: proliferating cell nuclear antigen

Blood test	Patient value	Reference range
Haemoglobin (g/L)	109	130–180
White cell count (× 10^9^/L)	5.8	4–11
Platelet count (× 10^9^/L)	359 × 109	150–400
Corrected calcium (mmol/L)	2.31	2.10–2.67
Phosphate (mmol/L)	1.06	0.75–1.50
Magnesium (mmol/L)	0.77	0.7–1.1
Sodium (mmol/L)	135	135–145
Potassium (mmol/L)	4.5	3.5–5.2
Chloride (mmol/L)	102	95–110
Bicarbonate (mmol/L)	22	22–32
Urea (mmol/L)	9.6	4–9
Creatinine (μmol/L)	716	60–110
Total bilirubin (μmol/L)	6	< 20
Total protein (g/L)	55	60–80
Albumin (g/L)	27	30–44
Globulin (g/L)	28	22–42
Alkaline phosphatase (unit/L)	73	30–110
Gamma-glutamyl transferase (unit/L)	42	5–50
Alanine transaminase (unit/L)	< 6	10–50
Aspartate aminotransferase (unit/L)	19	10–35
Lipase (unit/L)	48	10–60
Uric acid (mmol/L)	0.44	0.20–0.42
Thyroid stimulating hormone (mIU/L)	1.32	0.4–4.0
Lactate dehydrogenase (unit/L)	165	120–250
C-reactive protein (mg/mL)	86	< 5
C3 (g/L)	1.01	0.82–1.85
C4 (g/L)	0.09	0.15–0.53
Antinuclear antibodies	1:160 speckled pattern	–
Extractable nuclear antigens (SS-A/Ro, SS-B/La, RNP, Sm, Scl-70, Jo-1, PCNA, Ribosomal P and Pm-Scl)	Negative	–
Anti-neutrophil cytoplasmic antibodies	Not detected at 1:20	–

He had further hospitalisations for fever of unclear source, weight loss and ongoing abdominal pain. His inflammatory markers were raised: C-reactive protein > 200 mg/mL (ref < 5) and procalcitonin 2.75 ug/L (ref < 0.05). Abdominal imaging revealed new mild hepatosplenomegaly (liver 20 cm, spleen 14 cm). His serum immunoelectrophoresis demonstrated two trace kappa restrictions in the gamma region with no reactivity to IgG, IgA, IgM, IgD or IgE heavy-chain antisera. Peripheral blood flow cytometry revealed two B-cell populations: B cells which were polyclonal with a normal kappa:lambda (K:L) ratio (4% lymphocytes) and a light-chain restricted (kappa) B-cell population co-expressing CD45/11c/19/20/79b/81. This was suggestive of a CD11c + B-cell clonal lymphoproliferation likely non-Hodgkin’s lymphoma, either marginal zone or splenic lymphoma. Bone marrow biopsy showed moderate hypercellularity with trilineage hyperplasia, lymphocytes not increased (6% nucleated cells) and cytogenetics did not reveal any structural or numerical abnormalities.

Imaging at this time included a CT neck, chest, abdomen and pelvis scan that demonstrated discontinuous segments of bowel wall thickening involving the proximal-mid small bowel loops within the left side of the abdomen with free fluid and prominent mesenteric lymph nodes up to 11 mm. Whole-body gallium scan showed mild gallium accumulation in the small bowel loops in the left upper to mid-abdomen. Both transthoracic and transoesophageal echocardiograms did not reveal any valvular vegetations. A whole-body fluorodeoxyglucose (FDG) positron emission tomography (PET) scan was done to further investigate his ongoing fevers and showed non-specific sub-centimetre mesenteric and inguinal lymph nodes with increased uptake in the spleen and no evidence of vasculitis.

**Fig. 1:**
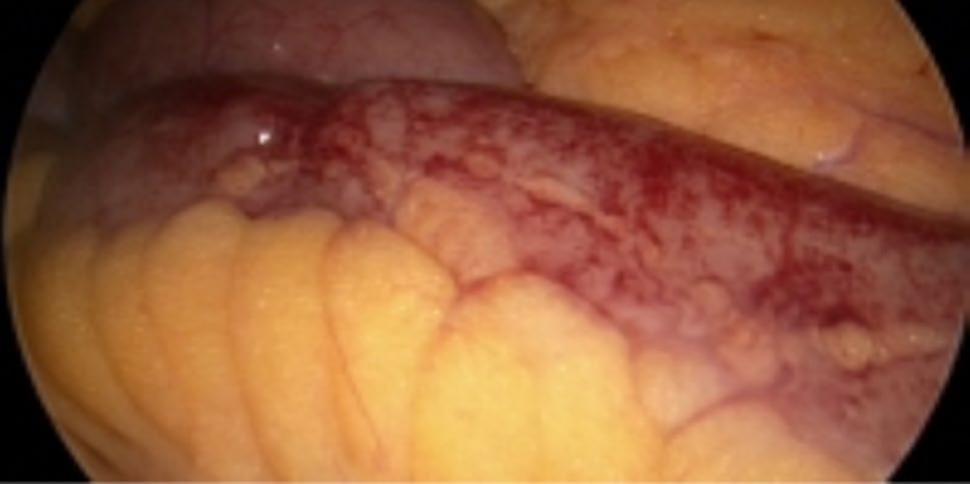
Diagnostic laparoscopy intra-operative images showing haemorrhagic serositis

A diagnostic laparoscopy was performed with the aim to obtain a lymph-node biopsy. Laparoscopy revealed haemorrhagic ascitic fluid and haemorrhagic serositis mostly involving the mid to distal small bowel (Fig. 1). Lymphadenopathy was seen in retroperitoneum and deep mesenteric nodes adjacent to duodenojejunal flexure; however, biopsy was not attempted given the risk of vascular injury was considered too high.

Given a tissue biopsy was not obtained on diagnostic laparoscopy, gastrointestinal endoscopy was pursued. His upper gastrointestinal endoscopy and colonoscopy found patchy mild inflammation characterised by shallow ulcerations in the jejunum and non-specific inflammation in the right colon with loss of vascular pattern and mild friability. Biopsies of the gastrum, jejunum, ileum and left and right colon were obtained. The jejunal biopsy revealed focal vasculitis involving a small muscularised blood vessel and a separate isolated small blood vessel with Periodic Acid-Schiff (PAS)-positive intraluminal material. Cytomegalovirus (CMV) immunohistochemistry was negative and there were no granulomas. The terminal ileum biopsy revealed mild focal acute inflammation. The gastric biopsies showed minimal patchy chronic inflammation. The colon biopsies were normal.

**Fig. 2:**
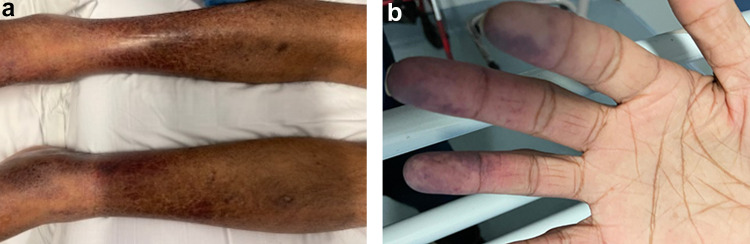
Lower limb skin rash (**a**) and digital ischaemia (**b**)

In addition, during the admission, he developed a bilateral non-blanching rash on his lower limbs (Fig. 2a) and cyanotic discolouration of his fingertips particularly the right second, third and fourth digits (Fig. 2b). The skin biopsy of his lower limb rash revealed leukocytoclastic vasculitis with partial epidermal necrosis and PAS-positive laminal thrombi in small vessels. He also developed generalised weakness of his arms and legs requiring a lumbar puncture that was unremarkable.

Testing for cryoglobulins revealed a type II cryoglobulinaemia consisting of an IgM kappa monoclonal band with polyclonal IgG (cryocrit 5%). His autoimmune blood tests at this time were as follows: C3 0.69 g/L (ref 0.82 to 1.85), C4 0.03 g/L (ref 0.15 to 0.53), rheumatoid factor 499 IU/mL (ref < 30), Hepatitis B and C antibodies negative, QuantiFERON gold negative, anti-cyclic citrullinated peptide (anti-CCP) antibodies < 1 U/mL (ref < 5), cardiolipin IgG < 3 U/mL (ref < 20) and cardiolipin IgM < 1 U/mL (ref < 20). He was also found to have a severe hypogammaglobulinaemia (IgG < 1.1 g/L [ref 7 to 16], IgA 0.72 g/L [ref 0.7 to 4], IgM 0.83 g/L [ref 0.4 to 2.3]) and was commenced on intravenous immunoglobulin replacement.

**Fig. 3:**
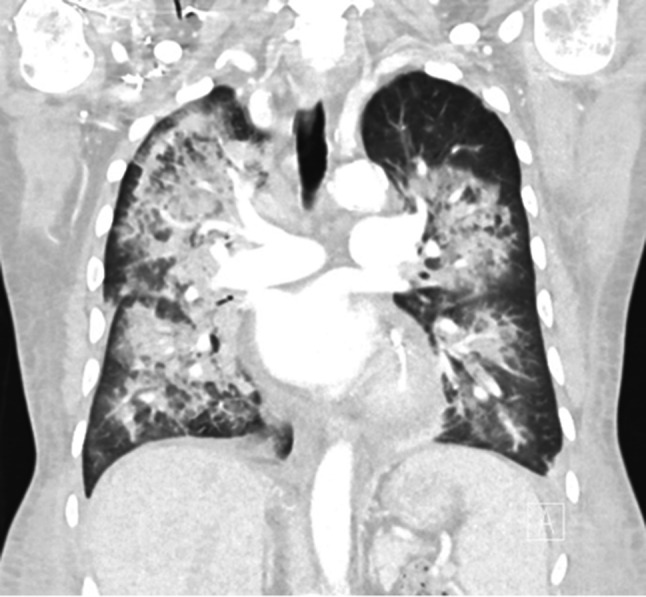
Computed tomography (CT) chest coronial image consistent with vasculitis-associated pulmonary haemorrhage

Given the above findings, he was diagnosed with a type II cryoglobulinaemia vasculitis with mainly cutaneous and gastrointestinal involvement. He was commenced on IV methylprednisolone 500 mg daily for 3 days (followed by oral prednisolone 75 mg daily on a weaning protocol) in addition to rituximab 375 mg/m2. His cryocrit reduced to 2% after treatment commencement. After three doses of rituximab, he developed haemoptysis and his CT chest showed extensive airspace consolidation in a perihilar distribution with sparing of the subpleural regions (Fig. 3). For further investigation, he underwent a bronchoscopy which showed bloodstained fluid that had negative Gram stain, scant polymorphs and was negative for acid fast bacilli, fungal infection, *Mycobacterium tuberculosis* and malignant cells. Respiratory virus polymerase chain reaction was negative for influenza A virus, influenza B virus, respiratory syncytial virus, parechovirus, human parainfluenza 1–4, human rhinovirus/enterovirus, metapneumovirus, adenovirus, bordetella and *Mycoplasma pneumoniae*. On blood tests, his cryocrit was 3%, anti-glomerular basement membrane antibodies (BIO-FLASH) was < 2.9 CU (ref < 20) and anti-neutrophil cytoplasmic antibodies (ANCA) were not detected at 1:20. Given the lack of another precipitating factor, the pulmonary haemorrhage was presumed to be cryoglobulinaemia vasculitis associated. This was successfully treated with five sessions of centrifugal plasma exchange separate to haemodialysis sessions and with substitution fluid warmed 4% albumin. After his final dose of rituximab therapy, his cryocrit improved to < 1% and C4 was 0.75 g/L (ref 0.15 to 0.53).

He has had no relapses of cryoglobulinaemic vasculitis since treatment and his marginal zone lymphoma is being monitored as an outpatient.

## Discussion

This patient presented a diagnostic dilemma, because he had a constellation of multi-organ involvement across multiple presentations. It is debatable if the initial presentation 3 years ago with leukocytoclastic vasculitis may have been the first sign of an underlying cryoglobulinaemia vasculitis. However, leukocytoclastic vasculitis is a histopathological finding with many differential diagnoses and can often be idiopathic [3]. It is also unclear whether the episodes of visual loss and the cerebral vascular calibre irregularities on imaging are related to the cryoglobulinaemia vasculitis.

The patient described in this case already had end-stage renal failure prior to diagnosis of cryoglobulinaemia vasculitis. This effectively precludes us from using renal manifestations to aid in the diagnosis of cryoglobulinaemia vasculitis and leaves us to focussing on the extra-renal manifestations in the diagnostic work up. In hindsight, we could have also tested for novel biomarkers such as serum soluble interleukin-2 receptor and it would be interesting to know whether that could have aided in the diagnostic workup [4].

Previous studies of patients on haemodialysis have found a higher incidence of cryoglobulins compared to healthy adults. Prior studies found that higher prevalence of Hepatitis C Virus (HCV) in patients on haemodialysis could partially explain the higher incidence of cryoglobulins; however, there was still a proportion of patients without HCV who had cryoglobulins [5–7]. Postulated reasons for the higher incidence of cryoglobulinaemia in patients on haemodialysis could be related to chronic inflammation and leukocyte dysfunction. One cross-sectional study found the cryocrit values amongst patients on haemodialysis to be lower compared to those patients who were not on haemodialysis [5].

Classically, mixed cryoglobulinaemia vasculitis is associated with HCV infection and in some cases, it can be related to B-cell lymphoproliferative disorders, autoimmune conditions and other infections [2]. Considering that our patient’s HCV (and other viral) serology was negative and his autoimmune screen was negative, it is possible that his cryoglobulinaemia vasculitis was associated with the newly diagnosed marginal zone lymphoma. Supporting this association would be that both disorders were characterised by the same monoclonal kappa IgM.

The pathophysiological mechanisms leading to mixed cryoglobulinaemia vasculitis are best understood for HCV-associated cryoglobulinaemia vasculitis and are less clear for other aetiologies [8]. In this case, there was likely an unknown trigger (potentially environmental or genetic) leading to B-cell lymphoproliferation that produced the detected cryoglobulins [9]. In mixed cryoglobulinaemia vasculitis, the B-cell lymphoproliferation can lead to a range of B-cell malignancies including diffuse large B-cell lymphoma (most commonly), marginal zone lymphoma (as in this case) and B-cell chronic lymphocytic leukaemia [9].

The cryocrit value is often measured in cryoglobulinaemia and refers to the precipitate’s percentage of the total volume [10]. The measurement of cryocrit values is not standardised between labs and the value itself is not known to correlate with disease severity or prognosis—there are patients with severe disease with low cryocrit values and patients with asymptomatic disease who have high cryocrit values [11]. However, the cryocrit value for a particular patient can be tracked to monitor progress and response to treatment, as was done in this case [10].

Alveolar haemorrhage is a rare complication of cryoglobulinaemia and generally has a poor prognosis [12]. In the work up of alveolar haemorrhage, other causes need to be first excluded such as infection, ANCA-associated vasculitis, Goodpasture syndrome, medications and malignancy. Cryoglobulinaemia can be considered the most likely cause of alveolar haemorrhage if no other aeitology is identified [13]. It is interesting to note that in our case, the patient’s cryocrit increased at the time of his pulmonary haemorrhage event (from cryocrit 2% to 3%).

Identifying the underlying predisposing disorder is essential for definitive treatment. In HCV-associated cryoglobulinaemia vasculitis, there is efficacy with antiviral therapy, particularly for mild cases of vasculitis [8]. Likewise, patients identified to have an underlying B-cell proliferative disorder or autoimmune disorder should receive directed therapy for that disorder. For severe cases of cryoglobulinaemia vasculitis, immunosuppression has traditionally been used for treatment. Multiple therapeutic regimens have been trailed including corticosteroids, azathioprine, alkylating agents and rituximab. Rituximab has emerged as the preferred immunosuppression agent based on its efficacy and tolerance profile [8]. There is limited role for the use of plasma exchange in the management of cryoglobulinaemia vasculitis unless there is symptomatic hyperviscosity or life-threatening manifestations such as pulmonary haemorrhage. Plasma exchange has also been trialled in cases of refractory cryoglobulinaemia vasculitis [8].

In conclusion, this patient’s constellation of symptoms was explained by a cryoglobulinaemia vasculitis associated with a B-cell lymphoproliferative disorder. The clinical features of cryoglobulinaemia may be difficult to detect in patients with end-stage renal failure on chronic haemodialysis patients and vigilance is required. Prompt treatment of cryoglobulinaemia vasculitis and underlying predisposing conditions can lead to improved recovery and outcomes. Further studies investigating a possible association between cryoglobulinaemia and haemodialysis are needed.
