# Correction: Mixed cryoglobulinaemia vasculitis secondary to marginal zone lymphoma in a patient with end‑stage renal failure on haemodialysis

**DOI:** 10.1007/s13730-026-01109-2

**Published:** 2026-04-22

**Authors:** Craig Peter Coorey, Amirhossein Aarabi, Karthik Kumar

**Affiliations:** 1 Gosford Hospital, Gosford, New South Wales, Australia; 2 School of Medicine, Western Sydney University, New South Wales, Australia

**Correction to: CEN Case Reports (2024) 13:168–173** 10.1007/s13730-023-00823-5

In the sentence beginning ‘His autoimmune’ in this article, the value ‘C3 0.69 g/L (ref 0.69 to 1.85)’ should have read ‘C3 0.69 g/L (ref 0.82 to 1.85)’.

The original article has been corrected.

